# *In Vitro* Plant Regeneration from Commercial Cultivars of Soybean

**DOI:** 10.1155/2017/7379693

**Published:** 2017-06-11

**Authors:** Ghulam Raza, Mohan B. Singh, Prem L. Bhalla

**Affiliations:** Plant Molecular Biology and Biotechnology Laboratory, Faculty of Veterinary and Agricultural Sciences, The University of Melbourne, Parkville, VIC 3010, Australia

## Abstract

Soybean, a major legume crop, is the source of vegetable oil and protein. There is a need for transgenic approaches to breeding superior soybean varieties to meet future climate challenges. Efficient plant regeneration is a prerequisite for successful application of genetic transformation technology. Soybean cultivars are classified into different maturity groups based on photoperiod requirements. In this study, nine soybean varieties belonging to different maturity group were regenerated successfully from three different explants: half split hypocotyl, complete hypocotyl, and cotyledonary node. All the genotypes and explant types responded by producing adventitious shoots. Shoot induction potential ranged within 60–87%, 50–100%, and 75–100%, and regeneration rate ranged within 4.2–10, 2.7–4.2, and 2.6–10.5 shoots per explant using half split hypocotyl, complete hypocotyl, and cotyledonary explants, respectively, among all the tested genotypes. Bunya variety showed the best regeneration response using half split and complete hypocotyl explants and the PNR791 with cotyledonary node. The regenerated shoots were successfully rooted and acclimatized to glasshouse conditions. This study shows that commercial varieties of soybean are amenable to shoot regeneration with high regeneration frequencies and could be exploited for genetic transformation. Further, our results show no correlation between shoots regeneration capacity with the maturity grouping of the soybean cultivars tested.

## 1. Introduction

Legumes, characterized by their ability to fix nitrogen, are of fundamental importance for agriculture systems. Soybean [*Glycine max* (L.) Merr.] is a major annual legume crop used for human and animal feed and source of vegetable oil globally. Soybean is a dietary staple for more than 1 billion people in many developing countries. In addition, soybean seeds are also a source of high-quality health benefit products such as antioxidants, omega-3 fatty acids, amino acids, phytoestrogens, and foliate. Soybean are also used in a variety of processed food products (soy milk, soy cheese, yogurt, and ice cream) and considered as nutritionally equivalent to meat. Soybean oil is used for many human food products and cooking oil and has numerous industrial applications including soaps, oils, paints, cosmetics, plastics, clothing, and solvents. Recently, soybean is also used as biofuel crop to meet global energy demands putting significant pressure on crop production for food. Moreover, global climate change presents threats to our food supply.

Soybean is a paleopolyploid with three rounds of whole genome duplication [1, 2]. Recently, there has been a significant effort in understanding the genome of soybean by undertaking large‐scale genome sequencing, microarray, expressed sequence tag sequencing, and transcriptome analyses ([3, 4]; reviewed in Liew et al. 2014). In addition, comprehensive studies on synteny, quantitative trait locus (QTL) mapping, and comparative genomics have increased our knowledge on soybean and closely related legumes of economic importance [5–8].

Soybean has a wide adaptability with a large number of cultivars showing variability to flower in response to day length and temperature. Thus, soybean cultivars have been divided into thirteen maturity groups based on the photoperiod and temperature requirements for flowering. However, our understanding of molecular basis of soybean maturity types remains unrevealed. Recent studies have reported association of nine loci (*E1, E2, E3, E4, E5, E6, E7*,* E8,* and* J*) with soybean maturity where* E1* has been shown to encode a B3 superfamily member [9, 10],* E2* encodes an ortholog of* Arabidopsis* GI [9, 11], and* E3* and* E4* encode the photoreceptors GmPHYA3 and GmPHYA2, respectively [12]. In addition, role of blue light receptors has been proposed for soybean distribution [13].

Soybean, originating in China, now is a major crop in North and South America and Asia. Soybean production has increased fivefold during the past four decades making it the major protein, and oil crop globally [1] and this growth are predicted to continue due to increasing demand for food, feed, and fuel. Significant efforts have been made to improve soybean through conventional breeding. However, classical breeding programs for soybean are limited by its self-pollination ability [14]. Therefore, there is a need for transgenic approaches to its improvement. Efficient plant regeneration protocol is the prerequisite for successful application of genetic transformation technology. There has been ongoing effort for enhancing the plant regeneration potential of soybean via either organogenesis or somatic embryogenesis. Organogenesis based regeneration is attractive due to an abundant and convenient supply of explants. Earlier reports have tried using different explants such as cotyledonary node [15, 16], whole cotyledonary node [17], epicotyl and primary leaves [18, 19], primary leaf nodes [20], and hypocotyls [14, 21–24].

In Australia, soybean has been commercialized relatively recently in early 1950 and now is grown across Australia; Queensland, New South Wales, and northern Victoria and western Australia. There are some varieties with various desirable traits grown in Australia. Bunya and A6785 varieties belong to maturity group VI and are grown in Queensland. Bunya variety is recently bred and released by the CSIRO for tofu markets, while A6785 variety is preferred for soymilk and soy flour. Bunya has very large seeds that are prone to damage at the harvesting time. Seed size of A6785 is smaller but has lower protein content. A6785 variety is considered drought tolerant by the Industry. Snowy is recommended for the Riverina region of NSW and northern Victoria. Snowy variety offers good yield and quality suitable for tofu and soy milk for human consumption. It is an early maturing variety bred for frost prone areas of Victoria and NSW. Fernside has flexible planting requirements with desired grain size making this variety suitable for human and animal consumption. Fernside is considered superior to A6785, Warrigal, and Jabiru varieties. Moonbi has low yield but has fast drying time for harvest. Due to photoperiod and temperature requirements for soybean flowering, adoption of varieties to different parts of Australia is restricted. Moreover, availability of suitable soybean varieties across all the potential growing regions of Australia is lacking. Hence, there is a need for breeding soybean varieties suitable for the environmental differences and agronomic and quality trait targets.

We aim to develop genetic transformation technology for commercial soybean varieties by identifying the most optimal way to regenerate shoots from different soybean explants and evaluate whether shoots regeneration capacity is related to the maturity grouping of the soybean cultivars. It is well established that crop plant regeneration ability is tissue and genotype dependent. Hence, we compared the regeneration ability of three different soybean explants from nine soybean cultivars. This is the first report of in vitro plant regeneration of commercially grown Australian soybean varieties. The protocol developed would assist in developing genetic transformation system for these soybean varieties.

## 2. Materials and Methods

### 2.1. Plant Material

Nine cultivars (Bunya, Fernside, Snowy, Moon B1, A6785, PNR791, Bragg, Jack, and William) of soybean (*Glycine max* (L.) Merr.) were used in this study. Bunya, Fernside, Moon B1, Snowy, A6785, and PNR791 cultivars were obtained from Maralong Pty Ltd., PB-Agrifood, Brisbane, Australia. Seeds of these cultivars were stored in airtight container at room temperature until used.

### 2.2. Seeds Sterilization

Seeds were surface-sterilized by exposing seeds to chlorine gas for 48 h. For this, seeds were put in 90 mm Petri plate and placed inside a desiccator along with a beaker containing 100 ml commercial bleach having 8–12% chlorine contents. Chlorine gas was generated by addition of 3.5 ml concentrated hydrochloric acid. After 48 h the seeds were taken out and rinsed with sterile distilled water for 5 times. The excess water was removed by placing the seeds on sterile filter paper. Seeds were cultured on germination medium for six days at 26 ± 2°C with 16 h day length and 8 h night. Germination medium comprised Gamborg B5 basal salts and vitamins [25], 100 mg/L my-inositol (I3011 Sigma-Aldrich), 585.6 mg/L 2-(N-morpholino)ethanesulfonic acid sodium [(MES) (M3058; Sigma-Aldrich)], 1 mg/L 6-benzylaminopurine [(BAP), (B3408; Sigma-Aldrich)], and 0.8% agar (A1296; Sigma-Aldrich).

### 2.3. Explants Preparation

Three different explants, half split hypocotyls, hypocotyls, and cotyledonary nodes, were excised from 6-day-old soybean seedlings. The cotyledonary nodes explants were obtained as described by [26] and complete hypocotyl explants were obtained as described by [27]. The half split hypocotyls were obtained by longitudinal sectioning of hypocotyls explants into two halves [21].

### 2.4. Shoot Induction and Elongation

The explants were cultured in three replicates (10 explants/replicate; 10 explants/Petri dish) on shoot induction medium. Shoot induction medium (SSIM) comprised Gamborg B_5_ basal salts with vitamins, 100 mg/L my-inositol, 585.6 mg/L MES, 1.67 mg/L BAP, and 0.8% agar. After 15 days the explants with induced shoots were further cultured on shoot elongation medium. Shoot elongation medium (SSEM) comprised MS basal salts with vitamins [28], 100 mg/L my-inositol, 100 mg/L L-asparagine (A4159; Sigma-Aldrich), 100 mg/L L-pyroglutamic acid (P5960, Sigma-Aldrich), 10 mg/L silver nitrate [(AgNO_3_), (7761-88-8; Aldrich Chem. Co.)], 1 mg/L zeatin (Z899; Phytotechnology), 0.5 mg/L gibberellic acid [(GA_3_), (G7645; Sigma-Aldrich)], 0.1 mg/L indole-3-acetic acid [(IAA), (102037; ICN Biomedicals Inc.)], 585.6 mg/L MES, and 0.8% agar.

After 15 days, the shoot induction data were recorded. The hypocotyl parts (1-2 mm) from complete hypocotyl explants were removed, and upper parts with a bunch of small shoots were further subcultured on SSEM. Similarly, from the cotyledonary node explants, the cotyledon and hypocotyl (1-2 mm) were removed, and the remaining parts were further cultured in a plastic jar containing SSEM while half split hypocotyl explants were subcultured on SSEM without any dissection for one month.

### 2.5. Root Initiation

Well-developed shoots of 3–5 cm were cultured on rooting medium. Rooting medium (RIM) comprised 1/2 strength B5 salts, full strength vitamins, 100 mg/L my-inositol, 585.6 mg/L MES, 1 mg/L indole butyric acid [(IBA) (I5386; Sigma-Aldrich)], and 0.8% agar (A1296; Sigma-Aldrich). Most of the shoots formed roots on rooting medium. The regenerated plantlets were transferred to pots using commercially available potting mix and acclimatized to glasshouse conditions.

### 2.6. Tissue Culture Conditions

All tissue culture media were adjusted to pH 5.6 with 1 N KOH, autoclaved at 121°C for 20 minutes. Filter sterilized growth hormones BAP, IAA, IBA, zeatin, GA_3_, and AgNO_3_ were added to the autoclaved medium as required. The medium was poured into Petri dishes (100 × 20 mm), except for root induction media, for which transparent sterile plastic glass (60 × 60 × 90 mm) was used. The culture plates and glasses were sealed using 3 M surgical tape and were incubated at 26 ± 2°C and day length was set to 16 h (General Electric cool white fluorescent tubes) producing 50–80 *μ*molm^−2^s^−1^ at Petri dish level.

### 2.7. Pollen Viability Test

Fluorochromatic reaction (FCR) was used to test pollen viability. For this, fresh pollen from tissue culture regenerated plants and plants grown from seeds were incubated in a solution of fluorescein diacetate (0.2 mg/mL in 10% sucrose, F7378, Sigma-Aldrich) before observing pollen viability under a microscope [29]. At least 10 flowers from each plant were tested for pollen viability.

### 2.8. Statistical Analysis

Statistical significance of the retrieved data was validated using the chi-square test at *P* = 0.01 and a 1-way analysis of variance at *α* = 0.05 by using statistical software (Genstat).

## 3. Results and Discussion

In this study, we evaluated plant regeneration from nine soybean genotypes using three different seedling explants; seedling explants used were half split hypocotyl, complete hypocotyl, and cotyledonary node. Seed germination medium used included cytokinin and BAP (1 mg/L). Our preliminary experiments using seed germination medium devoid of cytokinin showed low regeneration frequency (data was not shown), suggesting that cytokinin inclusion during seed germination enhances the shoots regeneration process. Seed germination on the cytokinin-containing medium resulted in seedlings with enlarged cotyledons, short and thicker hypocotyls, and small thicker roots devoid of auxiliary roots. No significant differences in seedlings growth and development among the genotypes tested except seedlings of William cultivar had comparatively longer hypocotyl. These observations are in agreement with Shan et al. [14] who noted similar developmental effects of cytokinin treatment on soybean seedlings. However, their study reported no effect on the development of auxiliary roots.

Within one week on shoot induction media, all explant types started to expand and swell; no difference in genotypes tested was observed. Subsequently, all explants started to show multiple shoot initials originating from the nodal ends of cotyledon explants, from the centre of half split hypocotyl explants while shoot initials were initiated from the whole upper surface of complete hypocotyl explants including nodal end. Cotyledonary node showed multiple shoot initials organised in a bunch but larger in size as compared to half split and complete hypocotyl explants. Among this bunch of small shoots regenerated from cotyledon explant, there was a dominant and well-developed thick, large single shoot and, in some cultivars, only this single shoot was observed.

The origin and the presence of multiple shoots were independent to maturity grouping of the soybean cultivars tested. For example, Bunya cultivar, belonging to maturity group VI, showed the presence of multiple shoot initials originating from all over the half split and complete hypocotyl explants. On the other hand, half split hypocotyl explants of cultivar A6785 (maturity group VI) and complete hypocotyl explants of Snowy (maturity group III) produced only 1-2 small shoot initials originating from the nodal end (Figure 3). The cotyledonary node of PNR791 (maturity group V) and Bunya (maturity group VI) produced a large cluster of multiple shoots while Jack (maturity group II) produced 1-2 shoots only (Figure 3).

Shoot regeneration potential of each explant type of different genotypes was recorded after 15 days of shoot initiation. The analysis of variance results showed highly significant differences (*P* < 0.01) among the response of genotypes as well as explant types for shoot initiation and regeneration. All the genotypes and explant types showed shoot primordia or shoot initials with variable induction potential ranging from 60 to 87% for half split hypocotyl explants, 50 to 100% for complete hypocotyl explants, and 75 to 100% with cotyledonary node (Table 1).

Comparison of regeneration frequency from different explants and genotypes tested showed a significant variation. The range in different cultivars was from 4.2 to 10.0, 2.7 to 4.2, and 2.6 to 10.5 shoots per explant using half split hypocotyl, complete hypocotyl, and cotyledonary explants, respectively (Table 1). These results indicated the better regeneration potential of half split hypocotyl and cotyledonary node compared to complete hypocotyl explants. However, when we compared half split hypocotyl and cotyledonary node, low range of shoot numbers (4.2) of half split hypocotyl explant was better compared to cotyledonary node (2.6).

The protocol developed in the study regenerated more shoots per explant using half split hypocotyl (4.2–10.0) explants compared to published report of regeneration efficiency of 1.0–5.0 shoots per explant [21]. It might be due to difference in regeneration strategy such as addition of cytokinin in germination medium and using of B5 basal medium for germination and shoot elongation medium in this study.

In this report, maximum average shoots of 10.5 per explant from cotyledonary node were obtained. However, regeneration strategy in this study could not be recommended for complete hypocotyl explants due to low regeneration frequency per explant except for cv. Bunya (MG VI), which showed reasonable regenerated average shoots of 4.2 per explants. A lot of small shoots initials were observed from complete hypocotyl explants of cv. Bunya (MG VI). These results support earlier observations that explant is a major determinant factor for soybean in vitro plant regeneration [30].

No significant differences in shoot induction potential were observed among the genotypes tested in this study indicating that maturity grouping of genotypes has no effect on regeneration. However, significantly variable response for shoot regeneration among all cultivars was observed with different explants types, which also showed no relationship with maturity group. A maximum number of shoots (105) were regenerated in cv. PNR791 (MG V) followed by cv. MoonB1 (MG V) with 93 shoots while a minimum of 26 shoots was regenerated in cv. Jack (MG II) using cotyledonary node explants (Figure 4). The half split hypocotyl explants of cv. Bunya (MGVI) generated significant number of shoots (100) followed by cv. MoonB1 (75) and minimum shoots (42) were noted in cv. A6785 (MGVI; Figure 4). Complete hypocotyl explants were found to be the least responsive for shoots regeneration for all cultivars. Bunya cultivar (MGVII) produced maximum shoots (42) followed by cv. Bragg (MGVI) from complete hypocotyl explants. On the other hand, cv. Snowy (MGIII) showed the least number of shoots (Figure 4). This is the first report on the regeneration potential of commercial cultivars Bunya, A6785, MoonB1, Fernside, PNR791, and Snowy grown in Australia. Varied plant regeneration capacity among soybean cultivars has been reported in previous reports ([16, 17, 21]; Texeira et al. 2011). However, our results are not in agreement with [16] who reported that all soybean genotypes responded uniformly when cotyledonary node was used as explant.

After dissecting the shoots from each explant type, the remaining parts of all examined cultivars were further subcultured to see further proliferation and regeneration capacity. After one month, a clear influence of the genotype and explant types on repeated proliferation was noted. Half split hypocotyl explants showed the best proliferation while the cotyledonary node explants showed the minimum. Half split hypocotyl explants of cv. Snowy (MG III) showed maximum proliferation followed by cv. Bunya (MG V) while cv. Jack (MG II) showed minimum proliferation (Figure 5(a)). From complete hypocotyl explants, cv. Bunya (MG VI) showed maximum proliferation while cv. William (MG III) showed a minimum proliferation (Figure 5(b)). Cultivar MoonB1 (MG V) showed maximum proliferation and minimum proliferation noted in cv. William (MG III) from cotyledonary explants (Figure 5(c)). This suggests that new meristematic cells were initiated again in repetitive way (repetitive organogenesis) after subculturing, resulting in more proliferation and regenerated shoots. This repetitive proliferation potential is genotype and explant dependent. Similar findings were reported by Shan et al. [14] in soybean; new meristematic cells are initiated in a repetitive way from multiple bud tissues at the cotyledonary node, resulting in high shoot multiplication rate.

*Agrobacterium*-mediated transformation is a preferred method for soybean transformation. The efficiency of* Agrobacterium*-mediated transformation of soybean is influenced by many factors such as* Agrobacterium* strain, plasmid type, infection and cultivation regime, and regeneration of viable shoots. Of these factors, regeneration is highly genotype dependent. Most commonly used soybean cultivar for transformation is Jack with inferior agronomical traits. Studies using commercial soybean varieties are limited due to being unamenable to in vitro regeneration. Moreover, the transformation protocols reported so far are relatively specific to the cultivar Jack and are not easily reproducible for varieties with superior agronomical traits. Therefore, in vitro regeneration of viable plants is considered as one of the limiting factors in the application of the genetic transformation to soybean improvement.

*In vitro* regeneration is influenced by many factors including culture medium composition, culture environment, explant source, and genotype. In addition, regeneration of commercial cultivars has been reported to be slow and inefficient. Transformation efficiency is highly dependent on the regeneration as not all the cells transformed by* Agrobacterium* lead to the recovery of viable plant regeneration. It is well established that there is a direct correlation with ease of in vitro regeneration with the recovery of transgenic plants. Two major pathways for soybean shoot regeneration have been reported: organogenesis and somatic embryogenesis. Direct organs are produced from the explant or callus during organogenesis, and this pathway has been most widely used for soybean regeneration. Various soybean explants that have been used to regenerate plants via organogenesis are stem node [31], primary leaf node [20], hypocotyl segments [22, 32], embryonic axes [33], half seed [34, 35], and cotyledonary node [14, 17, 36, 37]. However, the cotyledonary node remains the most preferred explants for soybean plant regeneration via organogenesis. But our study showed a differential response from the explants tested from different cultivars highlighting importance of explant type for soybean genetic transformation.

Plant growth regulators play a significant role in plant regeneration. BAP has been reported to improve regeneration and number of shoots from cotyledonary node explants [36]. Some studies reported the positive influence of pretreatment of soybean seeds with TDZ or BAP on the regeneration of shoots (Wright et al. 1986; [14, 22]). Recently, Verma et al. [35] reported differential shoot development response based on the concentration of TDZ used in the culture medium. Thus, both BAP and TDZ are effective cytokinin for shoot organogenesis in soybean. In the present study, we found inclusion BAP in the medium effective in all the soybean cultivars tested.

**Figure 1:**
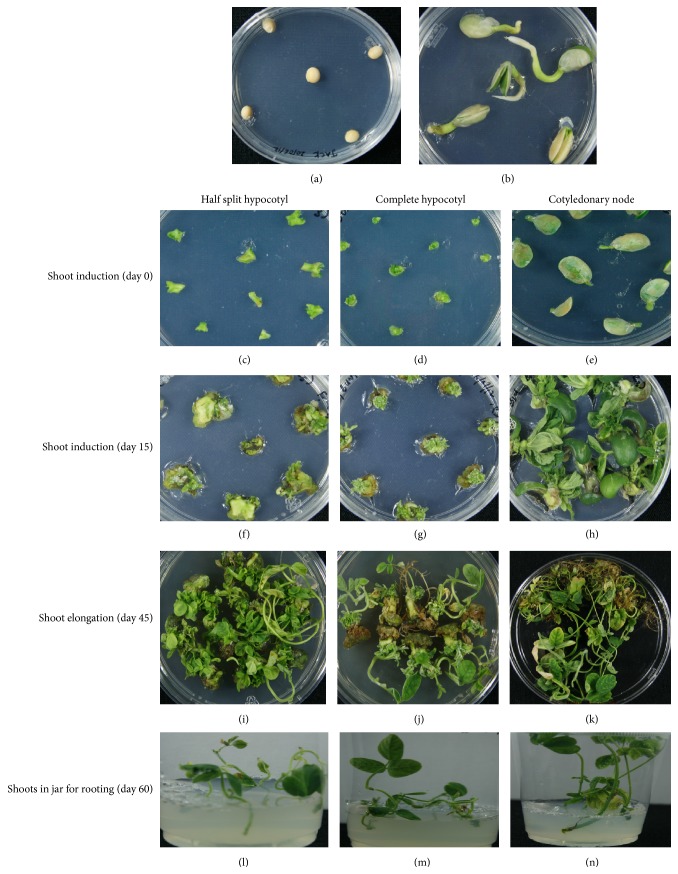
Tissue culture steps in development of soybean plants (cv. Bunya as a representative); (a) soybean seeds on germination medium; (b) 6-day-old seedlings; (c, d, e) half split hypocotyl, complete hypocotyl, and cotyledonary node explants on shoot induction medium (day 0); (f, g, h) half split hypocotyls, complete hypocotyls, and cotyledonary explants on shoot induction medium after 15 days; (i, j, k) regenerated shoots from half split hypocotyl, complete hypocotyl, and cotyledonary node explants after 45 days; (l, m, n) shoots from half split hypocotyl, complete hypocotyl, and cotyledonary node explants on rooting medium.

**Figure 2:**
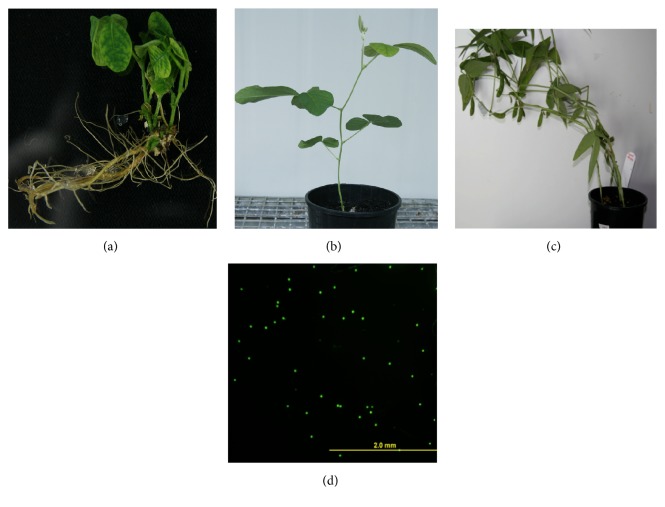
Normal plant growth of tissue culture plants (cv. Bunya as a representative): (a) rooted plant, (b) growth in soil, (c) pods development, and (d) viable pollens.

**Figure 3:**
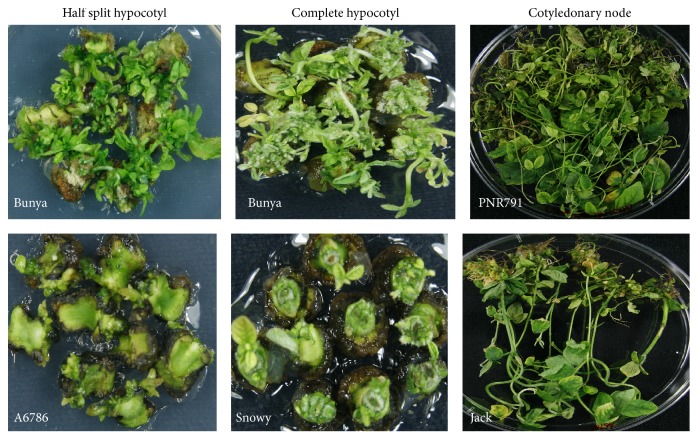
Cultivars showing maximum and minimum regeneration from three different explants types.

**Figure 4:**
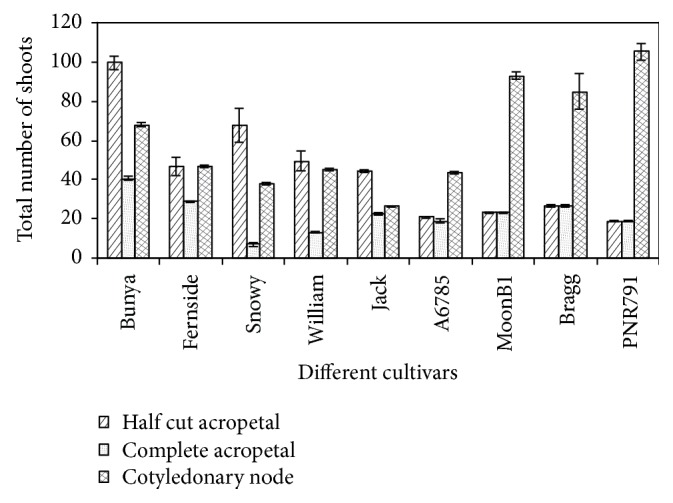
Regeneration response of nine soybean cultivars from three different explants types. Error bars indicate the standard error of mean (*n* = 3).

**Figure 5:**
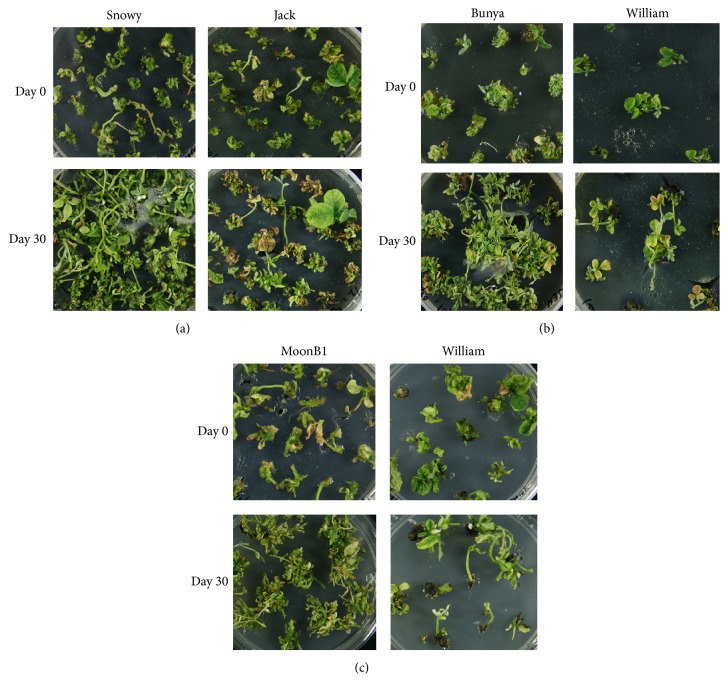
Cultivars showing maximum and minimum proliferation after one month of subculturing from three different explants types; (a) half split hypocotyls, (b) complete hypocotyls, and (c) cotyledonary node.

To conclude, in our studies plant regeneration was observed in all tested cultivars with all explants types.  Figure 1 shows different stages of plant regeneration from different seedling explants. The regenerated plants showed viable pollen and set seeds (Figure 2). No relationship of regeneration through organogenesis was observed with soybean maturity groups. Cultivar Bunya (MG VI) showed the best tissue culture response using half split and complete hypocotyl explants. This commercial cultivar could be an ideal candidate for genetic transformation. Cultivar PNR791 (MG V) was found to be the best for plant regeneration using cotyledonary node explants. The half split hypocotyl explants and cotyledonary explants type showed better plants regeneration and hence could be used for genetic transformation experiments.

## Figures and Tables

**Table 1:** Shoot regeneration from seedling explants of nine soybean genotypes.

Soybean cultivars	Maturity group	Explant type	Shoot induction (%)	Range/explant	Average shoots/explant
Jack	II	Cotyledonary node	83abcde	1–5	2.6
		Complete hypocotyl	50e	1–3	2.3
		Half split hypocotyl	73abcde	3–6	4.4

Snowy	III	Cotyledonary node	75abcde	1–5	3.8
		Complete hypocotyl	53de	0-1	0.7
		Half split hypocotyl	90ab	3–10	6.8

William	III	Cotyledonary node	100a	3–5	4.5
		Complete hypocotyl	64bcde	1-2	1.3
		Half split hypocotyl	72abcde	3–7	4.9

MoonB1	V	Cotyledonary node	100a	3–11	9.3
		Complete hypocotyl	73abcde	1–3	2.3
		Half split hypocotyl	87abc	3–10	7.5

PNR791	V	Cotyledonary node	100a	3–11	10.5
		Complete hypocotyl	73abcde	1-2	1.9
		Half split hypocotyl	73abcde	3–8	6.2

A6785	VI	Cotyledonary node	86abcde	1–6	4.4
		Complete hypocotyl	83 abcde	1-2	1.9
		Half split hypocotyl	77 abcde	3–6	4.2

Bunya	VI	Cotyledonary node	89 abcde	3–9	6.8
		Complete hypocotyl	100a	1–5	4.1
		Half split hypocotyl	86 abcde	8–12	10.0

Bragg	VII	Cotyledonary node	100a	3–10	8.5
		Complete hypocotyl	76abcde	1–3	2.7
		Half split hypocotyl	60cde	3–8	6.3

Fernside	VII	Cotyledonary node	82abcde	1–5	4.7
		Complete hypocotyl	97abcde	1–3	2.9
		Half split hypocotyl	77abcd	3–7	4.7
